# Tuberculosis clinical presentation and treatment outcomes in pregnancy: a prospective cohort study

**DOI:** 10.1186/s12879-020-05416-6

**Published:** 2020-09-18

**Authors:** Brittney J. van de Water, Meredith B. Brooks, Chuan-Chin Huang, Letizia Trevisi, Leonid Lecca, Carmen Contreras, Jerome Galea, Roger Calderon, Rosa Yataco, Megan Murray, Mercedes C. Becerra

**Affiliations:** 1 Department of Global Health and Social Medicine, Harvard Medical School, 641 Huntington Avenue, Boston, MA USA; 2 Partners In Health / Socios En Salud, Lima, Peru; 3 School of Social Work, University of South Florida, Tampa, FL USA

**Keywords:** Perinatal TB, Pregnancy, Epidemiology

## Abstract

**Background:**

There is limited research to guide TB treatment specifically in pregnant women and few studies have described the presentation of TB in pregnant women. We aimed to understand TB presentation and treatment outcomes in pregnant women in a low HIV burden setting. We describe a cohort of women of childbearing age treated for TB disease in Lima, Peru, and compare clinical presentation and treatment outcomes among pregnant and non-pregnant women between 2009 and 2012, including 36 pregnant women.

**Methods:**

This is a prospective cohort study. Subjects were recruited from across 106 public health centers in Lima, Peru. Baseline demographic, medical history, and drug-susceptibility test results were collected. We used descriptive statistics to describe demographic and clinical characteristics of the women using Pearson chi-squared, Fisher’s exact tests, or Kruskal-Wallis.

**Results:**

Among 4500 individuals with pulmonary TB disease, 1334 women were included in analysis with 36 (2.69%) pregnant women. Pregnant women had similar demographics, past medical histories, and clinical presentation to non-pregnant women, except being more likely to be married (*p* = 0.01) and have cardiac disease (*p* = 0.04) and less likely to have weight loss (*p* = 0.05). Twenty (71.4%) pregnant women had pan-susceptible TB compared with 616 (63.1%) non-pregnant women; four (14.3%) pregnant women had mono-resistant TB compared with 154 (15.8%) non-pregnant women; and four (14.3%) pregnant women had multi-drug-resistant TB compared with 140 (14.3%) of non-pregnant women (*p* = 0.53). Twenty-eight (96.6%) pregnant women had a successful outcome (cure, completed treatment, treatment ended early by clinical team) while one (3.4%) had an unsuccessful outcome (treatment failed) and 1074 (97.3%) non-pregnant women had a successful outcome while 30 (2.7%) had an unsuccessful outcome (*p* = 0.56).

**Conclusion:**

In this cohort with low HIV co-infection, we found high TB treatment success rates in both pregnant and non-pregnant women, irrespective of drug-susceptibility profiles. If treated appropriately, pregnant women with TB disease can have successful outcomes.

## Background

Globally each year, 3.2 million women become sick with tuberculosis (TB) [1]. In low- and middle-income countries, HIV/AIDS, maternal conditions, and TB account for nearly 50% of deaths among women in their reproductive years [1]. Additionally, with improvements in obstetrical care, non-obstetric causes including infectious diseases account for nearly 28% of maternal mortality worldwide [2]. Approximately 216,500 pregnant women were estimated to have TB in 2011, and it is not known how many pregnant women had drug-resistant TB (DR-TB) [3].

Diagnosing TB disease in pregnant women is challenging because some non-specific symptoms are common to both TB and pregnancy, such as shortness of breath and fatigue [4, 5]. However, when TB disease is diagnosed and treated, successful treatment outcomes have been seen in small cohorts of pregnant women [6]. Failure to treat TB during pregnancy increases risk of preventable death in both the woman and her child, thus TB treatment is recommended unless the risks outweigh the benefit of treatment [7].

Currently, first-line treatment for drug-susceptible TB is recommended during pregnancy [1]. First-line TB regimens— with isoniazid, rifampicin, pyrazinamide, and ethambutol— have been shown to be safe for pregnant women throughout all trimesters [8, 9]. Second-line drugs are also used during pregnancy, with more limited safety evidence [6, 10]. For example, aminoglycosides (such as kanamycin, amikacin, and streptomycin) should be avoided, especially within the first 20 weeks of pregnancy, due to the risk for ototoxicity and fetal malformation. Ethionamide and prothionamide can increase the risk of nausea and vomiting during pregnancy, thus these drugs are often avoided until after delivery [10]. However, most other second-line drugs are considered U.S. Food and Drug Administration class B (animal studies demonstrate no risk, no human studies) or C (animal studies demonstrate risk, no human studies), meaning they can be used in pregnancy without known adverse effects [10].

To our knowledge, pregnant women’s treatment outcomes have not been compared to non-pregnant women of the same age. We aimed to understand TB presentation and treatment outcomes in pregnant women in a low HIV burden setting. Thus, we describe a cohort of women of childbearing age treated for TB disease in Lima, Peru, and compared clinical presentation and treatment outcomes among pregnant and non-pregnant women. Additionally, we describe outcomes among women with DR-TB, because literature is scant among this sub-population. We hypothesized that pregnant and non-pregnant women will have similar presentations and treatment outcomes for TB disease.

## Methods

### Study setting

This is a sub-study of a prospective cohort study of household TB transmission conducted in Lima, Peru, described in detail elsewhere [11]. In brief, over a three-year period subjects were recruited in 106 public health centers across the study catchment area of approximately 3.3 million people. Between September 1, 2009 and August 29, 2012, individuals age 16 years and older who were diagnosed with pulmonary TB disease were invited to participate in the study (i.e., index subjects, *N* = 4500). After visiting recruited patients’ homes, consenting household contacts were enrolled as well. Study staff conducted interviews with index subjects to record baseline characteristics and also conducted follow-up interviews at two, six, 12, and 24 months. For patients with DR-TB, follow-up interviewers were also conducted at 36 and 48 months [11].

### Study design and study population

This is a descriptive secondary analysis of data from the cohort enrolled in the above parent study. Here we analyze the sub-set of women of childbearing age, which we defined as < 45 years. Sixteen years was the minimum age because only individuals > 15 years were enrolled as index subjects in the parent study [11].

### Sources of data and study measures

Baseline demographics and medical history were collected through an interview with a study staff member, and drug-susceptibility testing (DST) results for first- and second-line TB drugs were obtained through sputum collection and laboratory testing. Individuals were evaluated for TB disease with sputum smear microscopy with Ziehl-Neelsen staining and culture on solid Lowenstein-Jensen medium. In the absence of bacteriological confirmation of TB disease, a clinical diagnosis was based on evaluation by a medical doctor and radiography. Sputa were initially tested for drug sensitivity by using Lowenstein-Jensen medium while second-line DST was performed on Middlebrook 7H11 agar. Pregnancy was self-reported and confirmed with a urine test. Individuals with missing information on pregnancy status were excluded from analysis.

Outcomes were classified by local clinicians and extracted from the medical record. Outcomes were then linked to match 2013 WHO guidelines [12] for standardization of reporting, where possible. Composite outcomes are used; outcomes were categorized as either *successful* or *unsuccessful* (Table 1). We also report outcomes from individuals who were still on active treatment at the end of the study period, were lost to follow-up, or who were not evaluated for an outcome, but exclude them from the larger analysis. Subjects who were lost to follow up were excluded because it was impossible to determine whether they successfully completed treatment at another health facility outside of our catchment area or whether they experienced worsening of disease and, consequently, had an unsuccessful treatment outcome.

**Table 1:** Outcome variable definitions

Outcome	Composite Outcome	Definition (DS- definition / DR- definition)	Source	DS-, DR-, or Both
Cured	Successful	A pulmonary TB patient with bacteriologically confirmed TB at the beginning of treatment who was smear- or culture-negative in the last month of treatment and on at least one previous occasion. / Treatment completed as recommended by the national policy without evidence of failure AND three or more consecutive cultures taken at least 30 days apart are negative after the intensive phase.	WHO [12]	Both
Treatment Completed	Successful	A TB patient who completed treatment without evidence of failure BUT with no record to show that sputum smear or culture results in the last month of treatment and on at least one previous occasion were negative, either because tests were not done or because results are unavailable. / Treatment completed as recommended by the national policy without evidence of failure BUT no record that three or more consecutive cultures taken at least 30 days apart are negative after the intensive phase.	WHO [12]	Both
Treatment completed early	Successful	If the patient received more than 5 months of treatment (but less than 6 months) AND the outcome recorded by the treating physician was “completed dose” or “cured.”	Local physicians	DS-
Died	Unsuccessful	A TB patient who dies for any reason before starting treatment or during the course of treatment.	WHO [12]	Both
Treatment failed	Unsuccessful	A TB patient whose sputum smear or culture is positive at month 5 or later during treatment. / Treatment terminated or need for permanent regimen change of at least two anti-TB drugs because of: lack of conversion by the end of the intensive phase; bacteriological reversion in the continuation phase after conversion to negative; evidence of additional acquired resistance to fluoroquinolones; adverse drug reactions.	WHO [12]	Both
Clinical default	Unsuccessful	If the patient did not receive at least 18 months of treatment AND the outcome recorded by the treating physician was “cure.”	Local physicians	DR-
Lost to follow up	Lost to follow up	A TB patient who did not start treatment or whose treatment was interrupted for 2 consecutive months or more.	WHO^12^	Both
Not evaluated	Not evaluated	A TB patient for whom no treatment outcome is assigned. This includes patients “transferred out” to another treatment unit as well as patients for whom the treatment outcome is unknown to the reporting unit.	WHO^12^	Both
Still on active treatment	Still on active treatment	If no outcome was provided because the patient was still on active treatment.	Local physicians	Both

Cavity on chest x-ray was defined by radiologist reading. Isolates were classified as drug-susceptible if the drug-susceptibility test (DST) was susceptible to all drugs tested (including cycloserine, ethambutol, ethionamide, levofloxacin, pyrazinamide, isoniazid, rifampin, and streptomycin), while drug-resistant TB was defined as mono-, poly-, or multi-drug resistant. Age was self-reported. Marital status (married, divorced/separated, single, widowed), student status (student, not a student), education history (less than high school, high school or greater), medical history (cardiac disease, high blood pressure, asthma, kidney disease, diabetes, and history of TB) and clinical symptoms (cough, cough with blood, cough with phlegm, fever, weight loss, difficult breathing, and night sweats) were all self-reported. HIV status was confirmed with an ELISA blood test and Western Blot or indirect immunofluorescence assay [13]. Treatment delay was defined as the median number of days from symptom onset to TB diagnosis.

### Analysis

Categorical variables were reported as frequency and percent, and continuous variables were reported as median and interquartile range. To compare sociodemographic data, clinical symptoms, and TB treatment outcomes between pregnant and non-pregnant women, we used Pearson chi-squared, or Fisher’s exact tests for small cell sizes (fewer than five per cell). The Kruskal Wallis test was used to compare medians of the variable “age.” All analyses used SAS 9.4 (Cary, NC).

### Ethics review

All study participants provided voluntary, written informed consent prior to study participation. The Harvard School of Public Health (reference number 19332) and Peru’s Research Ethics Committee of the National Institute of Health provided Institutional Review Board approval.

## Results

**Fig. 1:**
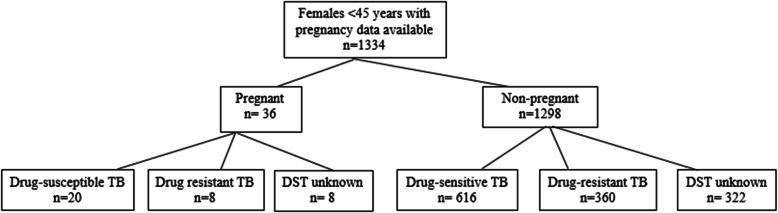
Participant flow diagram for inclusion in analysis

A total of 4500 individuals with pulmonary TB were enrolled in the study of whom 1368 were women between the ages of 16 and 45 years. Thirty-four women were excluded from analysis because their pregnancy status was unknown. Among the 1334 participants who met inclusion criteria (Fig. 1), 36 (2.7%) were pregnant; these had a median age of 24.5 (IQR 21–30.5) while 1298 (97.3%) non-pregnant women had a median age of 25 (IQR 20–32) (*p* = 0.9435) (Table 1). Most pregnant women were married (66.7%), were not students (94.4%), had less than a high school education (66.7%), were HIV-negative (97.1%), and on average, had low rates of comorbidities. Most demographics, past medical history, and symptoms among pregnant and non-pregnant women were not statistically different. However, pregnant women were more likely to be married (*p* = 0.01), have cardiac disease (*p* = 0.04), and have less weight loss (*p* = 0.05).

Drug-susceptibility testing (DST) data were available for 1004 (75.26%) women. Eight (22.2%) pregnant women had a clinical diagnosis of TB, similarly, 322 (24.8%) non-pregnant women had a clinical diagnosis of TB (*p* = 0.72). All other women had bacteriologically confirmed pulmonary TB. Twenty (71.4%) pregnant women had pan-susceptible TB compared with 616 (63.1%) non-pregnant women; four (14.3%) pregnant women had mono-resistant TB compared with 154 (15.8%) non-pregnant women, and four (14.3%) pregnant women had MDR-TB compared with 140 (14.3%) non-pregnant women (*p* = 0.53). Among the eight pregnant women with drug-resistant TB, two completed treatment, five were cured, and one was lost to follow up. Details of TB presentation, resistance profiles, and treatment regimens are reported in Table 2.

**Table 2:** Baseline characteristics of pregnant and non-pregnant women diagnosed with pulmonary tuberculosis

Characteristic	N	Pregnant	Non-pregnant	*p*-value
Age (Median (IQR))	1334	24.5 (21–30.5)	25 (20–32)	0.94^a^
Marital status	1325			0.01
Married		24 (66.7)	504 (39.1)	
Divorced/separated		0 (0.0)	47 (3.7)	
Single		11 (30.6)	734 (56.9)	
Widowed		1 (2.8)	4 (0.3)	
Student	1325	2 (5.6)	200 (15.5)	0.10
Education	1332			0.67
Less than HS		24 (66.7)	819 (63.2)	
HS or greater		12 (33.3)	477 (36.8)	
HIV positive	1321	1 (2.9)	28 (2.1)	0.54^b^
Cardiac disease	1330	3 (8.3)	27 (2.1)	0.04^b^
High blood pressure	1326	2 (5.7)	45 (3.5)	0.35^b^
Asthma	1330	4 (11.4)	120 (9.3)	0.56^b^
Kidney disease	1328	1 (2.9)	46 (3.6)	1.00^b^
Diabetes	1318	0 (0.0)	14 (1.1)	1.00^b^
History of TB	1331	6 (16.7)	155 (12.0)	0.43^b^
Microbiologically confirmed TB	1334	34 (94.4)	1179 (90.8)	0.77^b^
Treatment delay	1320	22 (14.0–48.5)	24 (13.0–41.5)	0.53^a^
Cavity on CXR	1304	5 (15.6)	348 (27.4)	0.14
Symptoms				
Cough	1334	34 (94.4)	1102 (84.9)	0.11
Cough with blood	1332	12 (33.3)	318 (24.5)	0.23
Cough with phlegm	1332	32 (88.9)	978 (75.5)	0.06
Fever	1332	8 (22.2)	379 (29.2)	0.36
Lost weight	1316	22 (61.1)	966 (75.5)	0.05
Difficulty breathing	1332	24 (66.7)	707 (54.6)	0.15
Night sweats	1330	20 (55.6)	658 (50.9)	0.58
Resistance pattern	1004			0.53
Pan-susceptible		20 (71.4)	616 (63.1)	
Mono-resistant		4 (14.3)	154 (15.8)	
Poly-resistant		0 (0.0)	66 (6.8)	
Multi-drug resistant +/−		4 (14.3)	140 (14.3)	

^a^ Kruskal-Wallis; ^b^ Fisher’s Exact

Among all women, 1319 (98.8%) had a treatment outcome reported (Table 3). Among pregnant women, 28 (96.6%) had a successful outcome (cure, completed treatment, treatment ended early by clinical team) while one (3.5%) had an unsuccessful outcome (treatment failed 5 months or later) (Table 4). One-thousand and seventy-four (98.4%) non-pregnant women had a successful outcome while 17 (1.6%) had an unsuccessful outcome (*p* = 0.38).

**Table 3:** Characteristics and outcomes of pregnant women with confirmed drug-resistant tuberculosis (*n*=8)

Participant	Age	TB Presentation	Baseline DST profile	TB treatment regimen	Treatment Outcome
1	19	• Smear +++ pulmonary TB • No history of TB • Cavity seen on CXR • Cough, cough with blood, cough with phlegm, weight loss, difficulty breathing	MDR (Resistant: INH, RIF, SM)	CS EMB ETH LEVO PZA	Treatment completed
2	21	• Smear + pulmonary TB • History of TB • Cough, cough with phlegm, difficulty breathing, night sweats	MDR (Resistant: INH, RIF, SM)	CS EMB ETH LEVO PZA	Lost to follow up
3	29	• Smear – pulmonary TB • No history of TB • Cough, cough with blood, cough with phlegm, difficulty breathing, night sweats	Mono-resistant (Resistant SM)	EMB INH PZA RIF	Cured
4	30	• Smear – pulmonary TB • No history of TB • Cough, cough with blood, cough with phlegm, weight loss, difficulty breathing, night sweats	Mono-resistant (Resistant: EMB)	EMB INH PZA RIF	Cured
5	34	• Smear ++ pulmonary TB • History of TB • Cough, cough with phlegm, weight loss	MDR (Resistant: PZA, INH, EMB, RIF, SM)	CS LEVO PAS PZA	Cured
6	17	• Smear ++ pulmonary TB • No history of TB • History of asthma • Difficulty breathing, night sweats	Mono-resistant (Resistant: SM)	EMB INH LEVO PZA	Cured
7	33	• Smear + pulmonary TB • History of TB • Cough, cough with phlegm, weight loss	Mono-resistant (Resistant: RIF)^a^	EMB INH PZA RIF	Cured
8	25	• Smear +++ pulmonary TB • No history of TB • Cough, cough with blood, cough with phlegm, weight loss	MDR (Resistant: INH, EMB, RIF, SM)	EMB INH PZA RIF	Treatment completed

*Abbreviations*: *CXR* chest x-ray, *CS* cycloserine, *EMB* ethambutol, *ETH* ethionamide, *LEVO* levofloxacin, *PZA* pyrazinamide, *INH* isoniazid, *RIF* rifampin, *SM* streptomycin

^a^RIF resistance was detected on a second drug susceptible test

**Table 4:** Successful versus non-successful outcomes among pregnant and non-pregnant women with pulmonary tuberculosis

	Pregnant women *n*=34	Non-pregnant women *n*=1285	*p*-value
Successful outcome	28 (96.6)	1072 (97.3)	0.56^a^
Cured	15 (44.1)	643 (50.1)	0.35
Treatment completed	12 (35.3)	400 (31.2)	0.75
Treatment ended early by clinical team	1 (2.9)	29 (2.3)	0.56^a^
Non-successful outcome	1 (2.9)	30 (2.7)	
Died	0 (0.0)	13 (1.0)	1.00^a^
Treatment failed	1 (2.9)	15 (1.2)	0.34^a^
Clinical default (DR-)	0 (0.0	2 (0.2)	1.00^a^
Censored	5 (14.7)	183 (14.2)	
On active treatment	1 (2.9)	54 (4.2)	1.00^a^
Lost to follow up	4 (11.8)	97 (7.6)	1.00^a^
Not evaluated	0 (0.0)	30 (2.3)	1.00^a^

^a^ Fisher’s Exact test

## Discussion

We observed similar clinical presentations and similar TB treatment outcomes between pregnant and non-pregnant women of childbearing age, regardless of the drug-resistance profile of the infecting strain. Pregnant women were more frequently married and, as expected, had less weight loss than non-pregnant women. Few other studies have reported on TB treatment outcomes among pregnant women, including DR-TB. One review found only nine published case reports describing DR-TB treatment during pregnancy; taken together those studies reported outcomes for a total of 73 women [6]. To our knowledge, no other studies have compared presentation or TB treatment outcomes among pregnant and non-pregnant women of childbearing age. Nor have other studies described presentation or symptomology of pregnant women with TB.

We found that pregnant women, regardless of drug resistance, can have successful treatment outcomes similar to non-pregnant women of childbearing age. Some studies have reported poor outcomes among pregnant women with TB [14, 15]; however, those focused on poor obstetric and infant outcomes, mostly in HIV co-infected women, and did not highlight TB treatment outcomes specifically. Another study, also in a largely HIV co-infected cohort, did highlight TB treatment outcomes, where 45% of the 73 women had unfavorable TB outcomes [16].

A recent systematic review of TB in pregnancy included 35 studies describing diagnosis, treatment, and follow-up [17]. Fourteen of these studies included pregnant women on TB treatment; 332/375 (88%) of women were cured. Four papers were specific to drug-resistant TB and pregnancy, reporting outcomes among a total of 55 women (one study including 38 women) with 42 being successfully treated (76%) [18–21]. Delay in treatment or loss to follow up were found to be the main causes of mortality and morbidity among mothers and infants. With appropriate treatment and close follow-up, pregnant women sick with TB can be cured and have positive maternal outcomes. Integration of TB screening programs with maternal care services could be an efficient way to detect cases in women that might otherwise remain undetected, as most TB in pregnancy is diagnosed in the third trimester of pregnancy or in the post-partum period [3, 4, 22, 23]. Another 2017 systematic review and meta-analysis including 13 studies found that TB disease in pregnancy is associated with adverse maternal and perinatal outcomes [7]. Our study adds that high rates of cure can be achieved in pregnant women treated for TB.

In addition to TB, HIV co-infection is also known to complicate pregnancy outcomes [6]. In this Lima cohort, only one pregnant woman was HIV co-infected [13]. However, much of the research on TB and pregnancy focuses on women with HIV or has been completed in settings with a high prevalence of HIV [5, 10, 14, 16, 18]. Many of the observed poor maternal and perinatal outcomes in those studies are due to HIV co-infection and not only to TB. Thus, our report adds to literature supporting good TB treatment outcomes among pregnant women without HIV co-infection.

There is a pressing need to expand evidence to optimize the delivery of DS- and DR-TB treatment for pregnant women [24]. Maternal health services could provide an important entry point to the healthcare system when women can be screened and treated for TB [24, 25]. If not diagnosed or treated early, there is a high risk of poor maternal and perinatal outcomes. Studies have reported a two-fold increase in premature birth and a six-fold increase in perinatal deaths in pregnant women who have delayed or interrupted TB treatment [7, 26]. WHO guidelines recommend using standardized regimens during pregnancy (including 2 months isoniazid, rifampicin, pyrazinamide, and ethambutol, followed by 4 months of isoniazid and rifampicin), and one study has shown these drugs to be non-teratogenic [9, 27]. Although evidence is limited, there is growing observational evidence that some DR-TB medications are safe during pregnancy [19, 20, 28]. Pregnant women should have prompt access to advances in TB treatment; however, due to the frequent exclusion of pregnant women from TB research studies, they are often a neglected population [24].

Notably, there is a dual benefit to treating a pregnant woman sick with TB: both to cure her and to eliminate the risk of her infecting her infant. The risk of transmission of TB can be high in the first 3 weeks of life; in the high HIV burden setting of Durban, South Africa, one study found that 15% of mothers transmitted TB to their infants [15]. Treating pregnant women directly protects infants, who face an exceedingly high risk of progression from TB infection to disease in the first year of life [29].

Our study had several limitations. First, the number of pregnant women in this cohort was relatively small, potentially making generalizability difficult. Second, few women in this cohort were HIV infected, thus making findings difficult to generalize in higher HIV burden settings. Additionally, we excluded some women from the primary analysis (successful/non-successful) if their TB treatment outcome was on active treatment, not evaluated, or lost to follow up. Had we included all of these outcomes as non-successful, we may have biased our results negatively. In addition, other studies have shown that most TB in pregnant women is diagnosed in the third trimester [22, 30]. We did not assess the trimester of pregnancy in this cohort of women. Finally, pregnancy and neonatal outcomes were not collected, thus we were unable to assess the health of the women and infants after TB treatment. Despite these limitations, this report provides evidence that pregnant women have a similar clinical presentation as do non-pregnant women and underscores that successful treatment outcomes among pregnant women with TB disease should be expected, regardless of the infecting strain’s drug-susceptibility profile.

## Conclusions

In sum, we found that pregnant women had similar rates of DS-TB and DR-TB as non-pregnant women, similar presentation, and similar treatment outcomes. Pregnant women can have successful treatment outcomes, regardless of the infecting strain. Further work is needed to understand how to leverage maternal care services to promote TB prevention, diagnosis, and treatment among pregnant women.

## Data Availability

The datasets analyzed during the current study are available from the corresponding author on reasonable request.

## Abbreviations

DST: Drug-susceptibility test
DS-TB: Drug sensitive tuberculosis
DR-TB: Drug resistant tuberculosis
ELISA: Enzyme-linked immunosorbent assay
HIV: Human immunodeficiency virus
MDR-TB: Multi-drug resistant tuberculosis
TB: Tuberculosis
